# Chemotherapy-induced neutropenia as a prognostic factor in advanced non-small-cell lung cancer: results from Japan Multinational Trial Organization LC00-03

**DOI:** 10.1038/sj.bjc.6605348

**Published:** 2009-09-29

**Authors:** Y Kishida, M Kawahara, S Teramukai, K Kubota, K Komuta, K Minato, T Mio, Y Fujita, T Yonei, K Nakano, M Tsuboi, K Shibata, S Atagi, T Kawaguchi, K Furuse, M Fukushima

**Affiliations:** 1 Department of Clinical Trial Design and Management, Graduate School of Medicine, Kyoto University, 54 Shogoin Kawahara-cho, Sakyo-ku, Kyoto 606-8507, Japan; 2 National Hospital Organisation, Kinki-chuo Chest Medical Centre, 1180 Nagasone-cho, Kita-ku, Sakai 591-8555, Japan; 3 National Cancer Centre Hospital, 5-1-1 Tsukiji, Chuo-ku, Tokyo 104-0045, Japan; 4 Osaka Police Hospital, 10-31 Kitayama-cho, Tennoji-ku, Osaka 543-0035, Japan; 5 Gunma Prefectural Cancer Centre, 617-1 Takabayashi-Nishicho, Ohta 373-8550, Japan; 6 Department of Multidisciplinary Cancer Treatment, Kyoto University Graduate School of Medicine, 54 Shogoin Kawahara-cho, Sakyo-ku, Kyoto 606-8507, Japan; 7 Dohoku National Hospital, 7-4048 Hanasaki-cho, Asahikawa 070-8644, Japan; 8 National Hospital Organisation, Okayama Medical Centre, 1711-1 Tamasu, Okayama 701-1192, Japan; 9 National Kure Medical Centre, 3-1 Aoyama-cho, Kure 737-0023, Japan; 10 Kanagawa Cancer Centre, 1-1-2 Nakao, Asahi-ku, Yokohama 241-0815, Japan; 11 Koseiren Takaoka Hospital, 5-10 Eiraku-cho, Takaoka 933-8555, Japan; 12 The Japan Multinational Trial Organisation, 474 Uehonnojimae-cho, Teramachi-Oike agaru, Nakagyo-ku, Kyoto 604-0925, Japan; 13 Translational Research Informatics Center, 1-5-4 Minatojimaminamimachi, Chuo-ku, Kobe 650-0047, Japan

**Keywords:** non-small-cell lung cancer, chemotherapy-induced neutropenia, overall survival, tumour response, landmark analysis

## Abstract

**Background:**

Neutropenia is a common adverse reaction of chemotherapy. We assessed whether chemotherapy-induced neutropenia could be a predictor of survival for patients with non-small-cell lung cancer (NSCLC).

**Methods:**

A total of 387 chemotherapy-naïve patients who received chemotherapy (vinorelbine and gemcitabine followed by docetaxel, or paclitaxel and carboplatin) in a randomised controlled trial were evaluated. The proportional-hazards regression model was used to examine the effects of chemotherapy-induced neutropenia and tumour response on overall survival. Landmark analysis was used to lessen the bias of more severe neutropenia resulting from more treatment cycles allowed by longer survival, whereby patients who died within 126 days of starting chemotherapy were excluded.

**Results:**

The adjusted hazard ratios for patients with grade-1 to 2 neutropenia or grade-3 to 4 neutropenia compared with no neutropenia were 0.59 (95% confidence interval (CI), 0.36–0.97) and 0.71 (95% CI, 0.49–1.03), respectively. The hazard ratios did not differ significantly between the patients who developed neutropenia with stable disease (SD), and those who lacked neutropenia with partial response (PR).

**Conclusion:**

Chemotherapy-induced neutropenia is a predictor of better survival for patients with advanced NSCLC. Prospective randomised trials of early-dose increases guided by chemotherapy-induced toxicities are warranted.

Chemotherapy is the standard remedy for patients with advanced cancer and neutropenia is an important dose-limiting toxicity of anticancer agents. Several studies since the late 1990s have reported that neutropenia (or leukopenia) that occurs during chemotherapy is a predictor of significantly longer survival for patients with breast cancer (Saarto *et al*, 1997; Cameron *et al*, 2003). A recent study by Di Maio *et al* (2005) confirmed the positive correlation between chemotherapy-induced neutropenia and increased survival in a pooled analysis of three randomised trials, which included 1265 patients with advanced non-small-cell lung cancer (NSCLC). Pallis *et al* (2008) have also shown the association between chemotherapy-induced neutropenia and better clinical outcome for patients with NSCLC. In a prospective survey of oral fluoropyrimidine S-1 in 1055 patients with advanced gastric cancer, Yamanaka *et al* (2007) reported that patients with moderate (grade-2) neutropenia had the longest survival.

In light of these reports, we have analysed the associations between the extent of chemotherapy-induced neutropenia, overall survival and tumour response by reviewing data from a clinical trial of patients with advanced NSCLC.

## Materials and methods

### Patients and treatment

A total of 401 chemotherapy-naïve patients with NSCLC stage IIIB (positive pleural effusion) or stage IV (no brain metastases), who had Eastern Cooperative Oncology Group (ECOG) performance status of 0 or 1, were enrolled in this randomised controlled trial (Japan Multinational Trial Organization LC00-03) between March 2001 and April 2005. Of 393 eligible patients, information regarding chemotherapy-induced neutropenia was not available for six patients. Thus, data from 387 patients were included in this analysis. These participants were divided into two groups by treatment. The experimental group (VGD arm, *n*=192) received three cycles of intravenous vinorelbine (25 mg/m^2^) and gemcitabine (1000 mg/m^2^) administered on days 1 and 8 of each 21 day cycle, followed by three cycles of single-agent intravenous docetaxel (60 mg/m^2^) administered on day 1 of each 21 day cycle. The standard regimen (PC arm, *n*=195) consisted of six cycles of intravenous paclitaxel (225 mg/m^2^) plus carboplatin (area under curve =6) infused on day 1 of each 21 day cycle. Details of dose modifications and reductions have been described previously (Kubota *et al*, 2008). The protocol permitted use of granulocyte-colony-stimulating factor (G-CSF) for patients with grade-3 neutropenia with fever or grade-4 leukopenia or neutropenia, but did not permit prophylactic use.

### Statistical analysis

Neutrophil counts were recorded on day 1, 8 and 15 in each treatment cycle for all patients and neutropenia was categorised using the National Cancer Institute common terminology criteria for adverse events (CTCAE, version 2.0). Tumour response was assessed by the Response Evaluation Criteria in Solid Tumors (RECIST) Group criteria. Overall survival was defined as time from randomisation until death from any cause. To evaluate the prognostic impact of chemotherapy-induced neutropenia, we first identified the worst grade of neutropenia during treatment for each patient. Then, using the proportional-hazards regression model, we estimated hazard ratios for overall survival according to the worst grade of neutropenia, after adjustment for covariates.

The participants in the trial had advanced NSCLC and a considerable number of patients died during the treatment period. This can lead to serious bias and result in a false-positive association between chemotherapy-induced neutropenia and longer survival, because patients who die during treatment receive fewer cycles of chemotherapy and, therefore, have less chance of developing more severe neutropenia. To lessen this bias, we used landmark analysis, whereby patients who died within 126 days (i.e., six 21-day cycles) of starting chemotherapy were excluded.

Survival curves were estimated using the Kaplan–Meier method. All reported p values are two-tailed; a value below 0.05 was considered statistically significant. All analyses were performed using SAS version 9.1 (SAS Institute, Cary, NC, USA).

## Results

### Incidence of neutropenia

Table 1 shows the grade of neutropenia according to treatment cycle of chemotherapy. A total of 275 of the 387 patients died. The median follow-up time for all patients was 393 days (range 19–1711). One hundred and fifty-five patients (40%) completed the planned six cycles of treatment and 308 patients (80%) had chemotherapy-induced neutropenia: 20 patients (5%) had grade 1, 38 (10%) had grade 2, 97 (25%) had grade 3 and 153 (40%) had grade 4 as the worst grade.

### G-CSF use

Table 2 shows the use of G-CSF according to the worst grade of neutropenia. Prophylactic use was not permitted. Nevertheless, G-CSF was administered to 15 patients who did not have grade-3 or greater neutropenia, or grade-4 leukopenia, so these patients were excluded from the analysis.

### Association between survival and chemotherapy-induced neutropenia

First, the association between the worst grade of neutropenia and the number of treatment cycles was evaluated. Patients who experienced more severe neutropenia received more cycles of chemotherapy (Table 3).

We then examined the causes of deaths that occurred within 126 days of the initiation of chemotherapy. Thirty-three patients died and lung cancer was the cause of death for 26 patients. Pneumonia, myocardial infarction, neutropenic sepsis and interstitial pneumonia resulting from previous radiation accounted for one death each. The causes of three deaths were unknown. Only one patient died from neutropenic sepsis through this clinical trial.

These data indicate that patients who had better outcomes could receive more cycles of treatment, resulting in higher incidence of chemotherapy-induced neutropenia. To lessen this bias, we used a landmark analysis, excluding the 33 patients who died and two patients who were lost to follow-up within 126 days of the initiation of chemotherapy. Thus, data from 337 patients were analysed: 162 patients in the VGD arm and 175 patients in the PC arm. Since the mean number of treatment cycles for patients who developed chemotherapy-induced neutropenia was still higher than that for patients who had no neutropenia (Table 3), we included the number of treatment cycles as a covariate in the multivariate analysis. Given the size of this trial, the patients were distributed into three categories according to the worst grade of neutropenia: absent (grade 0), mild (grades 1 and 2) and severe (grades 3 and 4).

**Figure 1:**
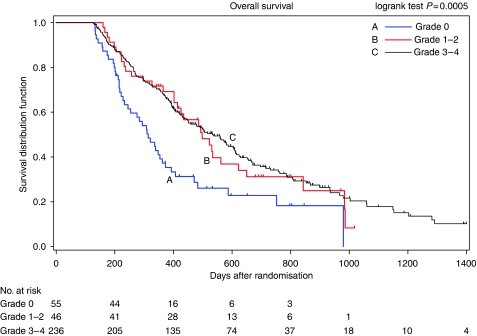
Kaplan–Meier survival curves according to the worst grade of chemotherapy-induced neutropenia (landmark time=126 days).

The median survival time was 10.5 months (95% confidence interval (CI) 8.2–12.4) for the grade-0 group (*n*=55), 16.6 months (95% CI 13.8–20.7) for the grade-1 to 2 group (*n*=46) and 17.8 months (95% CI 15.0–20.3) for the grade-3 to 4 group (*n*=236) (Figure 1). The baseline patient characteristics for the different groups are shown in Table 4. Using the proportional-hazards regression model to adjust for the imbalance of patient characteristics among groups, we estimated hazard ratios for overall survival according to the worst grade of neutropenia after adjustment for covariates (sex, smoking history, stage, ECOG performance status, weight loss, serum lactate dehydrogenase level, presence of bone, liver or skin metastases, pretreatment absolute neutrophil count and number of the treatment cycles as the known prognostic factors) (Paesmans *et al*, 1995; Pfister *et al*, 2004; Teramukai *et al*, 2009). Patients who had chemotherapy-induced neutropenia had lower risk of death than those who did not, although the difference between no neutropenia and grade-3 to 4 neutropenia was not significant. The adjusted hazard ratio compared with the grade-0 group was 0.59 (95% CI 0.36–0.97; *P*=0.036) for the grade-1 to 2 group, and that for the grade-3 to 4 group was 0.71 (95% CI 0.49–1.03; *P*=0.072) (Table 5). In both treatment arms, the proportion of patients who moved on from VGD or PC to second-line chemotherapy (e.g., because of progressive disease (PD)) was almost equal among the groups distributed by the grade of neutropenia.

We also estimated the hazard ratios for overall survival according to the combination of worst grade of neutropenia and best tumour response, after adjustment for the covariates listed above (Table 6). As a preliminary step, hazard ratios according to the best tumour response alone were calculated. The adjusted hazard ratio for stable disease (SD) compared with partial response (PR) as the best tumour response was 1.93 (95% CI, 1.39–2.67) and that for PD compared with PR was 3.31 (95% CI, 1.89–5.79). The adjusted hazard ratio compared with no neutropenia with PR was 0.29 (95% CI 0.11–0.80) for grade-1 to 2 neutropenia with PR; 0.44 (95% CI 0.21–0.92) for grade-3 to 4 neutropenia with PR; 0.78 (95% CI 0.33–1.87) for grade-1 to 2 neutropenia with SD and 0.80 (95% CI 0.38–1.70) for grade-3 to 4 neutropenia with SD. The hazard ratios did not differ significantly between the patients who developed neutropenia with SD and those who lacked neutropenia with PR.

## Discussion

It has been reported that haematological toxicity could be a measure of the biological activities of cytotoxic drugs. Many of us believe that administration of larger dose of chemotherapeutic agents over a defined period is more likely to result in success – the patient will have more chances to go into complete or partial remission, and this will improve survival (Luciani *et al*, 2009). However, several studies in the last decade have reported that larger doses of chemotherapy do not always improve prognosis (Stadtmauer *et al*, 2000; Möbus *et al*, 2007). Using a unique time-dependent approach to analyse data from a prospective survey of patients with advanced gastric cancer treated with oral fluoropyrimidine S-1, Yamanaka *et al* (2007) reported that survival was longest in patients who experienced grade-2 neutropenia as the worst grade.

Here we review data from a clinical trial of patients with advanced NSCLC. Patients who developed neutropenia showed longer survival than those who had no neutropenia. Furthermore, severe neutropenia (grade 3–4) was no better than mild neutropenia (grade 1–2) for prediction of overall survival. As a whole, these results are consistent with previous reports of the chemotherapy of NSCLC and gastric cancer (Di Maio *et al*, 2005; Yamanaka *et al*, 2007; Pallis *et al*, 2008), and strongly suggest that neutropenia *per se* is not important, but the use of neutropenia to reflect that an adequate dose has been given.

The dose of chemotherapeutic agents is usually determined on the basis of body surface area (BSA) or creatinine clearance; however, elimination of the agents will vary from patient to patient because of a variety of factors such as pharmacogenetic background (Friedman *et al*, 1999) and drug interactions (Relling *et al*, 2000). Variation in drug elimination may explain why some patients in this clinical trial experience severe toxicities or inadequate antitumour effects. Absence of neutropenia may mean that the doses of chemotherapeutic agents administered are not enough to produce the full antitumour effect. Gurney (2002) pointed out a poor correlation between BSA and the pharmacokinetics of anticancer agents (Newell, 2002).

From this perspective, this association also suggests that neutropenia or other toxicities induced by chemotherapy can be used as an indicator for planning regimens tailored to individual patients. When we administer chemotherapy to patients, we prepare a schedule for administration of each agent. Then, after initiation of chemotherapy, we often reduce the planned doses of agents in the event of severe neutropenia or other toxicities, whereas we seldom increase the dose if a patient lacks such toxicities. However, increasing the doses of agents to induce mild or moderate neutropenia may be of benefit for patients who do not show haematological or major non-haematological toxicities in the first or second cycle of treatment.

We have previously confirmed that increased pretreatment neutrophil count is an independent negative prognostic factor (Teramukai *et al*, 2009), and we included it as one of covariates in the present study. Tumour-related leukocytosis (neutrophilia) is encountered occasionally in patients with NSCLC and has recently been demonstrated to be an important negative prognostic factor for overall survival and time to progression in patients with NSCLC (Mandrekar *et al*, 2006). Although autonomous production of G-CSF and granulocyte–macrophage-colony-stimulating factor (GM-CSF) by tumour has been identified in some cases, leukocytosis (neutrophilia) in NSCLC patients is not fully understood and is likely to be caused by a combination of factors. Considering the negative prognostic value of leukocytosis (neutrophilia), it can be hypothesised that a proportion of the patients who do not develop neutropenia during treatment may have a poorer prognosis because they may be potentially affected by tumour-related leukocytosis (neutrophilia) and protected from chemotherapy-induced neutropenia (Maione *et al*, 2009). However, the results of our analysis suggest that chemotherapy-induced neutropenia is a predictor independent of NSCLC-related leukocytosis, since the risk of death estimated by the proportional-hazards regression model was significantly lower in patients who had grade-1 to 2 chemotherapy-induced neutropenia after adjustment for covariates, including pretreatment neutrophil count.

We estimated hazard ratios for the overall survival for subgroups assigned by the combination of the worst grade of neutropenia and the best tumour response. Patients who experienced neutropenia with PR as the best tumour response showed lower risk of death than those with PR who lacked neutropenia. The hazard ratios did not differ significantly between the patients who developed mild or severe neutropenia with SD and those with PR who lacked neutropenia. There are some limitations to the assessment of tumour size using the RECIST method or other widely used methods of assessing tumour response to anticancer therapy. Lara *et al* (2008) reported the importance of how to interpret SD and introduced the concept of disease control rate. Results from the randomised trial (JMTO LC00-03) and this study add further evidence that the association between the RECIST response and overall survival may depend on the grade of neutropenia and that the RESICT response may not be a surrogate endpoint for overall survival of advanced NSCLC in the chemotherapy setting (Kubota *et al*, 2008). Further investigation into this association in a large-scale meta-analysis would be helpful to resolve the important question of whether tumour response to anticancer agents could be used as a surrogate for overall survival in patients with advanced cancer (Ichikawa and Sasaki, 2006).

In conclusion, we confirm that chemotherapy-induced neutropenia can predict survival for patients with advanced NSCLC. This association also suggests the possibility that neutropenia, or other chemotherapy-induced toxicities, can be used as indicators in setting up dosage regimens that are tailored for individual patients. Categorisation of patients according to drug elimination capacity may be useful in determining initial dosage regimens, with subsequent fine-tuning depending on the presence or absence of haematological and non-haematological toxicities during early cycles. Prospective randomised trials of early-dose increases guided by chemotherapy-induced toxicities are, therefore, warranted.

## Figures and Tables

**Table 1:** The incidence of neutropenia according to treatment cycle (*n*=387). Values indicate number (%) of patients

**Treatment cycle**	**1**	**2**	**3**	**4**	**5**	**6**	**1–6**
**Number of patients**	**387**	**350**	**300**	**242**	**181**	**155**	**387**
Grade 0	140 (36)	123 (35)	113 (38)	90 (37)	73 (40)	81 (52)	79 (26)
Grade 1	26 (7)	31 (9)	26 (9)	16 (7)	11 (6)	4 (3)	20 (5)
Grade 2	42 (11)	50 (14)	30 (10)	33 (14)	19 (10)	18 (12)	38 (10)
Grade 3	89 (23)	87 (25)	79 (26)	53 (22)	43 (24)	30 (19)	97 (25)
Grade 4	90 (23)	59 (17)	52 (17)	50 (21)	35 (19)	22 (14)	153 (40)
Grades 1–4	247 (64)	227 (65)	187 (62)	152 (63)	108 (60)	74 (48)	308 (80)

**Table 2:** The use of G-CSF according to worst grade of neutropenia (*n*=387). Values indicate number (%) of patients

		**Use of G-CSF**	
**Worst grade of neutropenia**	***n***	**No**	**Yes**
Grade 0	79	70 (89)	9 (11)
Grade 1	20	19 (95)	1 (5)
Grade 2	38	33 (87)	5 (13)
Grade 3	97	65 (67)	32 (33)
Grade 4	153	25 (16)	128 (84)

Abbreviation: G-CSF, granulocyte-colony-stimulating factor.

**Table 3:** Association between worst grade of neutropenia and number of treatment cycles received

	**Number of treatment cycles**			
	**All patients (*n*=372)**		**Patients in landmark analysis (*n*=337)**	
**Worst grade of neutropenia**	***n***	**Mean±s.d.**	***n***	**Mean±s.d.**
Grade 0	70	3.4±1.9	55	3.9±1.9
Grade 1	19	4.0±1.7	18	3.9±1.7
Grade 2	33	4.2±1.9	28	4.6±1.7
Grade 3	97	4.5±1.6	90	4.7±1.5
Grade 4	153	4.5±1.7	146	4.6±1.6

**Table 4:** Baseline patient characteristics (*n*=337)

	**Grade 0 (*n*=55)**	**Grade 1–2 (*n*=46)**	**Grade 3–4 (*n*=236)**
*Age (years)*			
Median (range)	63 (45–78)	64 (43–81)	65 (33–81)
			
*Sex*			
Male (*n*) (%)	43 (78)	36 (78)	159 (67)
Female (*n*) (%)	12 (22)	10 (22)	77 (33)
			
*Smoking history*			
Current smokers (*n*) (%)	25 (45)	24 (52)	91 (39)
Former smokers (*n*) (%)	17 (31)	9 (20)	70 (30)
Non-smokers (*n*) (%)	11 (20)	10 (22)	65 (28)
Unknown (*n*) (%)	2 (4)	3 (7)	10 (4)
			
*NSCLC stage*			
IIIB (*n*) (%)	15 (27)	10 (22)	37 (16)
IV (*n*) (%)	40 (73)	36 (78)	199 (84)
			
*ECOG performance status*			
0 (*n*) (%)	16 (29)	16 (35)	112 (47)
1 (*n*) (%)	39 (71)	30 (65)	124 (53)
			
*Weight loss*			
< 5% (*n*) (%)	46 (84)	39 (85)	198 (84)
>5% (*n*) (%)	9 (16)	7 (15)	38 (16)
			
*LDH*			
Normal (*n*) (%)	40 (73)	32 (70)	172 (73)
High (*n*) (%)	15 (27)	14 (30)	64 (27)
			
*Bone metastases*			
No (*n*) (%)	42 (76)	36 (78)	170 (72)
Yes (*n*) (%)	13 (24)	10 (22)	66 (28)
			
*Liver metastases*			
No (*n*) (%)	52 (95)	43 (93)	217 (92)
Yes (*n*) (%)	3 (5)	3 (7)	19 (8)
			
*Skin metastases*			
No (*n*) (%)	54 (98)	45 (98)	233 (99)
Yes (*n*) (%)	1 (2)	1 (2)	3 (1)
			
*Pretreatment neutrophil count*			
Mean (per mm^3^±s.d.)	5828±2211	4968±1732	4427±2275

Abbreviations: ECOG=Eastern Cooperative Oncology Group; LDH=lactate dehydrogenase; NSCLC=non-small-cell lung cancer.

**Table 5:** Multivariate proportional-hazards regression analysis for associations between overall survival and worst grade of neutropenia (*n*=337)

	**Hazards ratio**	**95% CI**	***P*-value**
*Neutropenia*			
Grade 0	1	—	—
Grade 1/2	0.59	0.36–0.97	0.036
Grade 3/4	0.71	0.49–1.03	0.072
			
*Sex*			
Male	1	—	—
Female	0.75	0.53–1.06	0.104
			
*Smoking history*			
Non-/former smokers	1	—	—
Current smokers	1.67	1.23–2.28	0.001
			
*Stage*			
IIIB	1	—	—
IV	1.12	0.77–1.64	0.551
			
*Performance status*			
0	1	—	—
1	2.08	1.53–2.84	<0.0001
			
Weight loss			
<5%	1	—	—
⩾5%	1.06	0.74–1.50	0.765
			
*Serum LDH*			
Normal	1	—	—
High	1.64	1.20–2.25	0.002
			
*Bone metastasis*			
No	1	—	—
Yes	1.23	0.87–1.72	0.240
			
*Liver metastasis*			
No	1	—	—
Yes	1.62	1.02–2.60	0.043
			
*Skin metastasis*			
No	1	—	—
Yes	4.25	1.50–12.03	0.006
			
*Neutrophil count*			
< 4500/mm^3^	1	—	—
⩾4500/mm^3^	1.56	1.18–2.05	0.002
			
*Number of treatment cycles*			
1	1	—	—
2	0.94	0.48–1.84	0.866
3	1.07	0.58–1.96	0.838
4	0.88	0.48–1.61	0.674
5	0.59	0.29–1.21	0.151
6	0.58	0.34–1.01	0.054

Abbreviations: CI=confidence interval; LDH=lactate dehydrogenase.

**Table 6:** Multivariate proportional-hazards regression analysis for overall survival according to the worst grade of neutropenia and tumour response

	**Best tumour response**		
**Worst grade of neutropenia**	**Partial response (PR)**	**Stable disease (SD)**	**Progressive disease (PD)**
Grade 0	1	1.08 (0.47–2.48)	1.04 (0.33–3.25)
	(*n*=12)	(*n*=25)	(*n*=7)
Grade 1/2	0.29 (0.11–0.80)	0.78 (0.33–1.87)	2.05 (0.60–7.03)
	(*n*=16)	(*n*=23)	(*n*=5)
Grade 3/4	0.44 (0.21–0.92)	0.80 (0.38–1.70)	1.56 (0.63–3.88)
	(*n*=93)	(*n*=95)	(*n*=23)
			
Grade 0–4	1	1.93 (1.39–2.67)	3.31 (1.89–5.79)
	(*n*=121)	(*n*=143)	(*n*=35)

Abbreviation: CI=confidence interval.

Values indicate hazards ratios (95% CIs).
